# Rise and Growth of Vaccination Law.—I

**Published:** 1903-09-26

**Authors:** J. E. R. Stephens

**Affiliations:** Barrister-at-law, author of "Digest of Public Health Cases," etc., etc.


### RISE AND GROWTH OF VACCINATION LAW.—I.

By J. E. R. Stephens, Barrister-at-law, author of "Digest of Public Health Cases," etc., etc.

Vaccination (from Latin *vacca*, a cow) was the name given in France to the Jennerian practice of cow-poxing, shortly after the practice began in England (1799). The procedure was based almost exactly on the earlier practice of inoculating the small-pox, the matter being inserted under the skin of the arm by a lancet point; also the continuance of the same stock from arm to arm through a series of cases was an idea taken from some of the more adroit variolators. To replace small-pox inoculation by cow-pox inoculation under certain specified circumstances was Jenner's tentative project. Edward Jenner was born at Berkeley in Gloucestershire on May 17, 1749. His father, the Rev. Stephen Jenner, Rector of Rockhampton and Vicar of Berkeley, came of a family that had long been established in that county, and was possessed of considerable landed property. Jenner received his early education in local schools at Wotton-under-Edge and Cirencester, where he already showed a strong taste for natural history. The medical profession having been selected for him, he began his studies under Mr. Ludlow, a surgeon of Sodbury, near Bristol; but in his twenty-first year he proceeded to London, where he became a favourite pupil of the celebrated John Hunter, in whose house he resided for two years. During this period he was employed by Sir Joseph Banks to arrange and prepare the valuable zoological specimens which he had brought back from Captain Cook's first voyage in 1771. He must have acquitted himself satisfactorily in this task, since he was offered the post of naturalist in the second expedition, but declined it as well as other advantageous offers, preferring rather to practise his profession in his native place, and near his eldest brother, to whom he was much attached. His speedy success in practice did not engross his intellectual activity. He was the principal founder of a local medical society, to which he contributed several papers of marked ability, in one of which he apparently anticipated later discoveries concerning the rheumatic inflammation of the heart. He maintained a correspondence with John Hunter, under whose direction he investigated various points in biology, particularly the hibernating of hedgehogs and the habits of the cuckoo; his paper on the latter subject was laid by Hunter before the Royal Society, and appeared in the "Philosophical Transactions" for 1788.

Meanwhile the discovery that was to immortalise his memory had been slowly maturing in his mind. When only an apprentice at Sodbury his attention had been directed to the relations between cow-pox and small-pox in connection with a popular belief which he found current in Gloucestershire as to the antagonism between these two diseases. During his stay in London he appears to have mentioned the matter repeatedly to Hunter, who, being engrossed by other important pursuits, was not so strongly persuaded as Jenner was of its possible importance, yet spoke of it to his friends and in his lectures. After he began to practise in Berkeley, Jenner was always accustomed to inquire what his professional brethren thought of it; but he found that, when medical men had noticed the popular report at all, they supposed it to be based on an imperfect induction of facts. His first careful investigation of the subject dates from about 1775, and five years elapsed before he had succeeded in clearing away the most perplexing difficulties by which it was surrounded. He first satisfied himself that two different forms of disease had been hitherto confounded under the term "cow-pox," only one of which protected against small-pox, and that many of the cases of failure were to be thus accounted for; and his next step was to ascertain that the true cow-pox itself only protects when communicated at a particular stage of the disease. At the same time he came to the conclusion that "the grease" of horses was the same disease as cow-pox and small-pox, each being modified by the organism in which it was developed. For many years, cow-pox being scarce in his county, he had no opportunity of inoculating the disease, and so putting his discovery to the test, but he did all he could in the way of collecting information and communicating what he had ascertained. Thus in 1788 he carried a drawing of the cow-pox, as seen on the hands of a milkmaid, to London, and showed it to Sir E. Home and others, who agreed that it was "an interesting and curious subject," but by no means realised its practical importance. At length, on May 14th, 1796, he was able to inoculate James Phipps, a boy of about eight years old, with cow-pox matter. On the first of the following July the boy was carefully inoculated with variolous matter, but, as Jenner had predicted, no small-pox followed. The discovery was now complete, but he desired to act without precipitation, and was unable to repeat his experiment until 1798, owing to the diaappearance of cow-pox from the dairies. He then repeated his inoculations with the utmost care, and prepared a pamphlet which should announce his discovery to the world. Before publishing it, however, he thought it well to visit London, so as to demonstrate the truth of his assertions to his friends; but he remained in London nearly three months without being able to find any person who would submit to be vaccinated. Soon after he had returned home, however, Mr. Cline, an eminent surgeon, inoculated some vaccine matter over the diseased hip-joint of a child, thinking the counter-irritation might be useful, and found the patient afterwards incapable of acquiring small-pox. In the autumn of the same year, Jenner met with the first opposition to vaccination.

Early in 1799 a great series of public vaccinations was begun in London. In 1801 Dr. Jenner stated that "upwards of 6,000 persons had been inoculated with the virus of cow-pox, and that the far greater part of them had since been inoculated with that of small-pox, and exposed to its infection in every rational way that could be devised, but without effect." This statement was confirmed subsequently by other observers, notably by Dr. Woodville, of the Small-pox Hospital. In 1802 the subject of Dr. Jenner's discovery was brought before the Legislature, and a committee of the House of Commons, after examining a number of witnesses of the highest character and most extensive experience in the profession, reported in full corroboration of Jenner's views.

As the practice of vaccination increased, opposition grew up, and assertions of the wildest kind were made against it. In 1805, and again in 1806, inquiries were instituted as to the nature and truth of those assertions. The second and more important of these inquiries was undertaken by the Royal College of Physicians, in consequence of an address to the King, voted by the House of Commons. This inquiry lasted nine months, and the report of the College was decidedly in favour of the system. "The College of Physicians," it stated, "feel it their duty strongly to recommend the practice of vaccination. They have been led to this conclusion by no preconceived opinion, but by the most unbiassed judgment, formed from an irresistible weight of evidence which has been laid before them." The result was brought before the House of Commons on July 29th, 1807. "Henceforth," says Mr. Simon, "the public mind was apparently quite satisfied on the subject; and from this period begins to date the almost universal vaccination of children of the educated classes in this country." But although the practice of vaccination began to be popular in 1807, and although the House of Commons recognised its value, and rewarded its discoverer, it was not until the year 1840 that the matter was dealt with by legislative enactment.

Towards the end of 1802 steps were taken to form a society for the proper spread of vaccination in London, and the "Royal Jennerian Society" was finally established. This institution began very prosperously, more than 12,000 persons having been inoculated in the first eighteen months, and with such effect that the deaths from small-pox, which for the latter half of the eighteenth century had averaged 2,018 annually, fell in 1804, to 622. Unfortunately the chief resident inoculator soon set himself up as an authority opposed to Dr. Jenner, and this led to such dissensions as caused the society to die out in 1808. The Royal Jennerian Society having failed, the National Vaccine Establishment was founded, for the extension of vaccination in 1808.

*To be continued.*

